# Impact of sociodemographic factors on the consumption of tubers in Brazil

**DOI:** 10.1186/s12937-021-00709-1

**Published:** 2021-06-09

**Authors:** Justyna Horodyska, Sandrine Pigat, Jasmin Wonik, Foteini Bompola, David Cai, Colin D. Rehm, Tanhia D. Gonzalez

**Affiliations:** 1 Creme Global Ltd, Dublin, Ireland; 2 PepsiCo, Inc., Plano, TX USA; 3 PepsiCo, Inc., Purchase, New York, USA

**Keywords:** Dietary survey, BNDS, Intake, Vegetable, Mandioca, Cassava, Yam, Carrot, Potato, Sweet potato, Beet

## Abstract

**Background:**

Although tubers play a significant role in Brazilian agriculture, very little is known about the intake of tubers among the Brazilian population. The objective of this study was to characterize the intake of tubers across Brazil. The types of tubers consumed were quantified, and the impact of geographic and sociodemographic factors was assessed.

**Methods:**

This cross-sectional study is based on dietary intake data of 33,504 subjects obtained from the Brazilian National Dietary Survey. All tuber containing foods were identified, and the contribution of different tubers to overall tuber consumption in Brazil was quantified. Descriptive analyses assessed the impact of macroregion and sociodemographic characteristics on tuber consumption, and differences in intake were assessed using statistical tests. Lastly, the dietary intakes of tuber consumers and non-consumers were compared after adjusting for energy and covariates to determine if there were any major differences in dietary intakes between the two groups.

**Results:**

Fifty-five percent of the Brazilian population consumed tubers, which differed by macroregion. The intake of tubers among consumers also differed between macroregions. Overall, rural areas reported significantly higher mean daily intakes of tubers (122 g/day) among tuber consumers than urban areas (95 g/day). Mandioca and potato were the most commonly consumed tubers (59 and 43% prevalence, respectively, on any of the 2 days), while the highest daily intakes amongst tuber consumers across Brazil were noted for sweet potato (156 g/day) and potato (95 g/day). On a macroregion level, among tuber consumers, mandioca had the highest prevalence of consumption in the North (94%), Northeast (83%), and Central-West (68%), while consumption of potatoes was most prevalent in the Southeast (63%) and South (62%). Compared to women, small but significantly higher tuber intakes were noted for males (108 vs. 85 g/day). There were no significant differences in intakes among income quintiles. After adjusting for energy and other covariates, nutrient intakes between tuber and non-tuber consumers were not meaningfully different, with the exception of sodium (+ 6.0% comparing non-tuber to tuber consumers), iron (+ 6.1%), zinc (+ 5.7%), vitamin C (+ 8.3%), riboflavin (+ 9.0%), and folate (+ 7.9%).

**Conclusions:**

Tuber consumption is influenced by regional and sociodemographic characteristics of the Brazilian population. When looking at energy-adjusted nutrient intakes, diets of tuber consumers have resulted in somewhat lower intakes of some micronutrients, namely riboflavin, folate, vitamin C, iron, sodium, and zinc.

**Supplementary Information:**

The online version contains supplementary material available at 10.1186/s12937-021-00709-1.

## Introduction

Tubers play a significant role in Brazilian agriculture. The average per capita amount of roots and tubers produced in Brazil is 288 g/capita/day [1], potatoes, cassava, sweet potatoes, and yams being the main tuber crops grown in Brazil. Tubers are also an important part of the Brazilian diet and are often consumed as a side dish or bread substitute using tapioca. Cassava, also known as mandioca, is an important crop in Brazil, and it is often consumed whole, as part of cooked stews, and in the form of grits or flour, as a side dish [2]. Cassava flour is incorporated into traditional Brazilian dishes such as ‘Farofa’ and ‘Tutu de Feijao,’ while Cassava starch is used for making a traditional dish called ‘Pao de Queijo.’

Roots and tubers are primarily composed of water, carbohydrates, and low levels of protein and fat. The main carbohydrate found in tuber crops is starch. Polysaccharides that constitute the fiber component of roots and tubers include cellulose, hemicellulose, pectin, and resistant starch. When consumed with skin, potatoes and sweet potatoes can provide 2.1 g/100 g and 3.3 g/100 g of dietary fiber, respectively. Cooking with subsequent cooling of potatoes has shown to double their total amount of resistant starch [3]. Potatoes are a good source of Vitamin C (15% DV/110 g) and Vitamin B_6_ (13% DV/110 g), while sweet potatoes are an excellent source of Vitamin A (> 127% DV/110 g) as well as a good source of Vitamin B_5_ and B_6_ (12% DV/110 g and 11% DV/110 g, respectively). Yams, also known in Brazil as inhame, are a good source of Vitamin C (15% DV/110 g).

Although Brazil has been a large vegetable producer, with 40% of the production being roots and tubers, very little is known about the intake of tubers among the Brazilian population [4, 5]. The objective of this study was to characterize tuber consumption across all macroregions of Brazil.

## Materials and methods

### Data preparation and subjects

This study was based on the Brazilian National Dietary Survey (BNDS), which is a two-day non-consecutive 24-h record (including weekend days) of food and beverage consumption carried out by the Brazilian Institute of Geography and Statistics (IBGE) as part of the 2008–2009 Household Budget Survey. Briefly, participants provided all foods and beverages, including the time and place of eating occasions and cooking methods. Quality control was carried out by the interviewers while collecting data, and the data were entered into software developed by IBGE [4]. For the purpose of this study, 33,504 subjects between the ages of 10–104 years old (inclusive) with data necessary for the creation of survey weights were selected. A subset of this cohort, including only those who consumed one or more tuber containing foods/beverages on one or two of their recall days (i.e., tuber consumers), was created (*n* = 18,901). Other than their ages and survey weights, the following variables were collected: urban vs. rural residence, the region of residence, monthly household income, and sex.

### Food categorization

The dietary records were available for every subject in BNDS, which listed foods by name and the quantity consumed. The nutritional information for each food and beverage consumed was also available in BNDS. Foods consumed in the survey were identified from their names only as containing mandioca, yam, carrot, potato, beet, and sweet potato, which will be referred to herein as tuber types and collectively, tubers. From the 1971 unique foods in the BNDS, 162 foods were deemed to include tubers in-part or in-whole and different levels of processing (e.g. cooked cassava, cassava cake or Tapioca pudding) (Table S1). To account for the tuber part only within a composite dish consumed, information regarding the fraction of tuber was sourced from equivalent dishes/recipes from the United States Department of Agriculture What We Eat in America Food Commodity Intake Database 2005–10 (WWEIA-FCID, http://fcid.foodrisk.org/recipes/) and were applied (Table S1). For foods in BNDS for which no equivalents were available in the FCID, online research for recipes and Brazilian supermarket information was used to estimate a fraction. Where two or more different tubers were found to be part of a single food, each relevant fraction of the food was listed in the appropriate category. The methods of preparation/processing of the tuber portion of the food were also estimated from the food name only. These categories were tuber juice, tuber starch, whole tuber, whole tuber flour, a by-product of tuber processing and not applicable (uncategorized).

### Intake assessments

Nutrient intakes from each eating event were calculated as follows: the weight of food/beverage consumed in each eating event multiplied by nutrient concentration in that food/beverage. They were then summed up per person, per day, per nutrient. These values were divided by the number of consumption days to get an average daily nutrient intake over the two-day survey period per person. Where a given tuber type did not represent the entire food, only the fraction of the food which had been assigned to that tuber type was considered. However, the nutrient content of the complete dish (accounting for food ingredients other than tubers) was considered when calculating nutrient intakes. A total population’s tuber consumption and daily nutrient intake were represented to account for the survey weightings. The population’s consumption of each tuber type by weight (g) and nutrient intakes from the eating events was expressed as distributions using a Monte-Carlo simulation-based dietary exposure model [6] in Expert Models Food Data Science (Creme Global, Dublin, Ireland; accessed October 2017).

### Statistical analyses

Brazil is divided into five macroregions by the Brazilian Institute of Geography and Statistics (IBGE): North (7 states, largest metropolitan area Manaus), Northeast (9 states, Recife), Central-West (3 states. Brasilia), Southeast (4 states, Sao Paulo), and South (3 states, Porto Alegre). In this study, an analysis of variance (ANOVA) test was conducted to determine significant differences (*P* < 0.01) in the consumption of tubers and tuber types between rural and urban areas within Brazil. Furthermore multiple regression analysis was conducted to determine significant differences (*P* < 0.05) in the consumption of tubers and tuber types across the five Brazilian macroregions, as well as rural and urban areas within the macroregions. Multiple regression was also carried out to evaluate associations between tuber consumption and gender/age/income groups of the consumers across Brazil. Associations between the groups were deemed statistically significant at a *P* < 0.05. Post hoc Tukey test was performed to determine which pairs of the regional and sociodemographic group means were significantly different. Nutrient analysis was carried out in a regression model with adjustments for age, gender, region, rural/urban area, and income. Differences in mean intakes (g/day) normalized by energy (kcal/day) between tuber consumers and non-tuber consumers across Brazil were considered significant at a *P* < 0.01. All statistical analyses were performed using R (version 3.5.2, https://www.r-project.org) and Python (version 3.7.7, http://www.python.org).

## Results

The intake of tubers among tuber consumers in rural and urban areas across the five Brazilian macroregions is presented in Table 1. Fifty-five percent (18,901 people) of the total Brazilian population consumed at least one tuber containing food during the two-day survey period. Across all Brazil, rural and urban areas contained 58 and 54% tuber consumers, respectively. Tuber consumers in rural areas across Brazil ate significantly more tubers (mean: 122 g/day) as part of their diets than those in urban areas (mean: 95 g/day). Fifty-one percent of people in the Southeast consumed tubers compared to 55% in the Northeast macroregion. Of all macroregions, the highest tuber consumption among tuber consumers was noted in the South (mean: 112 g/day), followed by the North (mean: 107 g/day). Tuber intakes in these regions were significantly (*P* < 0.05) higher than in the Southeast and Central-West. The lowest intakes of tubers across all macroregions were found in the Central-West (mean: 91 g/day) and the Northeast (99 g/day). The tuber consumption in these regions was significantly (*P* < 0.05) lower than in the North and South. Differences in the tuber intakes between urban and rural areas were found across all macroregions, with significantly higher (P < 0.05) intakes noted in rural areas. Specifically, the North rural area consumed the highest amounts of tubers (mean: 149 g/day), while the Central-West urban area consumed the lowest amounts of tubers (mean: 85 g/day).

**Table 1:** Intake of tubers in Brazil according to regional and sociodemographic characteristics

Regions	Number of Tuber Consumers	Intake by Tuber Consumers (g/day)			Prevalence of tuber consumers (%)
		Median	Mean	SD	
All Brazil	18,901	78	101	102	54.7
Female	10,070	72	95^b^	93	54.7
Male	8831	80	108^a^	110	54.7
10–12 years	1182	66	91^b^	103	54.1
13–18 years	2415	72	97^b^	107	50.7
19–34 years	6073	80	104^b^	103	54.2
35–49 years	4646	80	103^b^	100	57.1
50–65 years	3045	75	104^b^	103	56.8
66+ years	1540	80	100^b^	93	52.2
Rural	4807	95	122^a^	127	58.1
Urban	14,094	74	95^b^	95	54
Income - 1st quintile^a^	3251	80	104^a^	110	51.7
Income - 2nd quintile^a^	3537	80	107^a^	112	51.1
Income - 3rd quintile^a^	3905	80	100^a^	97	52.8
Income - 4th quintile^a^	4087	80	105^a^	110	55.9
Income - 5th quintile^a^	4121	71	93^a^	87	59
North	3404	80	107^b^	115	73.1
Rural	1137	102	149^a^	154	80.3
Urban	2267	71	93^b^	87	70.4
Northeast	6872	70	99^b^	107	54.6
Rural	1737	80	116^a^	130	57.7
Urban	5135	66	95^b^	95	53.4
Southeast	3889	80	103^b^	93	51.4
Rural	754	95	115^a^	100	48.2
Urban	3135	80	96^b^	92	51.7
South	2332	88	112^b^	105	55.8
Rural	602	102	132^a^	112	60.5
Urban	1730	84	104^b^	103	54.9
Central-West	2404	53	91^b^	103	54.3
Rural	577	80	115^a^	121	46.1
Urban	1827	51	85^b^	101	55.4

Means were adjusted for age, gender, region, rural/urban area, and income. Means that do not share the same letter are significantly different (*p* < 0.05) to each other; SE: standard deviation; ^a^ Household monthly income (R$)

Differences in mean consumption between genders and age groups across all tuber consumers in Brazil were observed. Male consumers were significantly (*P* < 0.05) associated with increased intake of tubers. In relation to age there were no significantly different intakes observed. Differences in mean intakes between income groups across all tuber consumers in Brazil were not foundbut intakes tended to be higher in the lower income quintiles (the first quintile being lowest vs. fifth quintile being highest income group).

The intake of specific tuber types among tuber consumers in rural and urban areas across all Brazilian macroregions is shown in Table 2. Mandioca and potato were the most prevalent tubers in Brazil (59 and 43%, respectively). Consumption of mandioca was more prevalent amongst consumers in rural than in urban areas (76 and 55%, respectively). There were twice as many potato consumers in urban than in rural areas (47 and 24%, respectively). Sweet potato and yam were the least commonly consumed tubers in Brazil (3.8 and 3.5%, respectively). Sweet potato consumption was lower in urban areas than in rural areas (3.3 and 6.2%, respectively), while yams consumption was less disparate (3.3 and 3.5%, respectively). However, among consumers of each type, the highest daily intake across all of Brazil was noted for sweet potato, followed by potato and yam (mean: 156, 95, and 84 g/day, respectively).

**Table 2:** Intake of tuber types in rural and urban areas across Brazilian macroregions

Region	Mandioca		Potato		Sweet Potato		Carrot		Yam		Beet	
	% consuming	g/day among consumers	% consuming	g/day among consumers	% consuming	g/day among consumers	% consuming	g/day among consumers	% consuming	g/day among consumers	% consuming	g/day among consumers
All Brazil	59	77	43	95	3.8	156	11	30	3.5	84	4.6	50
Rural	76	110***	24	99***	6.2	191	5.6	34	3.3	90	2.4	57
Urban	55	67***	47	95***	3.3	141	12	29	3.5	83	5.1	49
North^£^	94	101^a^	14	79^c^	0.5	119^a^	3.7	24^a^	0.6	102a	1.1	62^ab^
Rural	96	143^a^	8	81^a^	0.2	131^a^	1	16^a^	1.5	116^a^	0.6	124^a^
Urban	93	84^b^	16	75^b^	0.6	107^a^	4.9	20^a^	0.2	75^a^	1.3	44^b^
Northeast^£^	83	78^c^	16	72^c^	8	172^a^	3	24^a^	7.8	82^c^	1.3	92^a^
Rural	88	96^a^	7.5	62^a^	9.4	206a	1.3	27^a^	4.2	84^a^	0.3	104^a^
Urban	81	73^b^	19	74^a^	7.5	159b	3.6	22^a^	9.2	82^b^	1.7	81^a^
Southeast^£^	42	58^d^	63	99^a^	1.6	135^b^	15	30^a^	3	87^bc^	5.9	40^b^
Rural	54	78^a^	55	105^a^	3.4	121^a^	9.1	40^a^	5.5	84^a^	4.2	46^a^
Urban	41	52^b^	64	98^a^	1.4	122^a^	15	30^a^	2.8	84^a^	6	42^a^
South^£^	34	88^bc^	62	104^a^	4.6	137^b^	21	29^a^	0.1	73^c^	8.4	45^b^
Rural	44	108^a^	56	110^a^	7.8	131^a^	19	26^a^	0	0	8.1	43^a^
Urban	32	74^b^	64	100^a^	3.9	121^a^	22	29^a^	0.1	66	8.4	41^a^
Central-West^£^	68	75^b^	35	76^b^	2.9	99^b^	13	28^a^	0.9	87^ab^	7.3	64^b^
Rural	76	109^a^	28	68^a^	2.1	134^a^	10	22^a^	3.3	167^a^	4.9	62^a^
Urban	67	67^b^	36	77^a^	3	98^a^	14	27^a^	0.6	38^b^	7.6	56^a^

^£^Means were adjusted for age, gender and income. Significant difference (**P < 0.01, ***P < 0.001) between macroregions/urban and rural areas within macroregions; Means that do not share the same letter are significantly different (p < 0.05) to each other; % of prevalence amongst consumers

On a macroregion level, the most commonly consumed tubers in the Northeast were mandioca (83%), potato (16%), and sweet potato (8%), while in the Southeast, the most popular tubers were potato (63%), mandioca (42%) and carrot (15%). The most commonly consumed tubers in the South, which is the third most populated region concentrating 15% of the Brazilian population [7], were potato (62%), mandioca (34%), and carrot (21%). The highest intakes of mandioca (mean:101 g/day) were observed in the North, while the highest intakes of potato were noted in the South (mean: 104 g/day). For sweet potato and beet, the highest consumption was found in the Northeast (mean:172 and 92 g/day, respectively). Lastly, the highest intakes of carrot were observed in the Southeast (mean: 30 g/day). Concerning differences in the consumption of tuber types among tuber consumers in rural and urban areas (Table 2), statistical analysis revealed that increased mandioca consumption was significantly (*P* < 0.05) associated with rural areas across all five macroregions. Moreover, potato intakes in the North rural area were significantly (P < 0.05) higher than those in the urban areas.

**Fig. 1:**
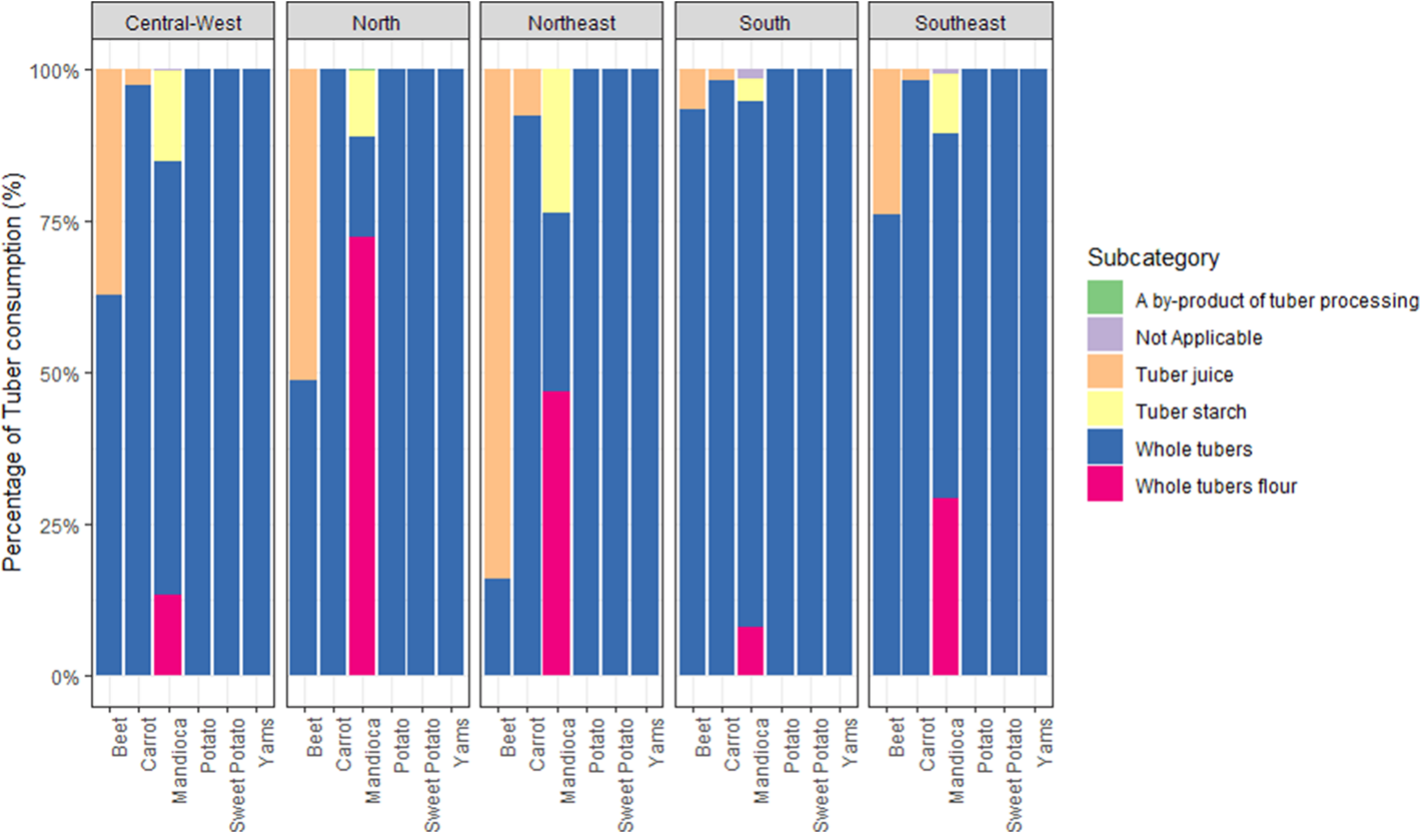
Percentage of tuber consumption per processing type across the five Brazilian macroregions

Regional differences in the preparation methods of tubers are shown in Fig. 1. This analysis aimed to explore tuber consumption habits in terms of the preparation methods amongst consumers living across the Brazilian macroregions. Mandioca was consumed in cooked dishes either whole and in the form of flour or starch, with the highest proportion of mandioca flour being consumed in the North, followed by Northeast and Southeast. Potato, sweet potato, and yam were consumed in dishes that incorporate the whole tuber across all five macroregions. Beet was consumed whole and as a juice, with the highest proportion of juice being in the Northeast region, followed by North and Central-West. While carrot was primarily consumed as a whole, a small proportion of juice consumption was observed in the Northeast, Southeast, South, and Central-West.

Nutrient intake of tuber consumers and non-tuber consumers across Brazil is shown in Table 3. Differences in energy intake (kcal/day) were significantly (*P* < 0.001) associated with tuber consumption, where individuals that consumed tubers had 16% higher energy intake per day when compared to non-tuber consumers. The regression analysis adjusted by sociodemographic variables also identified small but significant differences among tuber and non-tuber consumers in nutrients intakes.

**Table 3:** Nutrient intake of tuber consumers and non-tuber consumers across Brazil

Nutrient (unit)	Mean Intakes (SE)		Δ Mean Intakes (%)
	Tuber Consumers N = 18,901	Non-Tuber Consumers ***N*** = 14,603	
**Energy (kcal/day)**	1848 (0.003)	1600 (0.003)	248 (15.5)***
**Macronutrients**			
Protein (g/day)	83.3 (0.003)	75.5 (0.004)	7.8 (10.3)***
Protein (g/1000 kcal)	45.4 (0.002)	47.4 (0.002)	2 (4.2)***
Total Fat (g/day)	57.5 (0.004)	50.1 (0.004)	7.4 (14.8)***
Total Fat (g/1000 kcal)	30.8 (0.002)	30.9 (0.002)	0.09 (0.3)
Carbohydrates (g/day)	247 (0.003)	209 (0.003)	37.2 (17.7)***
Carbohydrates (g/1000 kcal)	134 (0.001)	132 (0.001)	2.3 (1.7)***
Total Fiber (g/day)	21.1 (0.004)	18.4 (0.004)	2.7 (14.6)***
Total Fiber (g/1000 kcal)	11.7 (0.003)	11.8 (0.003)	0.07 (0.6)***
**Micronutrients**			
Calcium (mg/1000 kcal)	277 (0.004)	285 (0.004)	8.3 (2.9)***
Magnesium (mg/1000 kcal)	139 (0.002)	140 (0.003)	0.7 (0.5)
Manganese (mg/1000 kcal)	2.94 (0.030)	3.06 (0.034)	0.12 (3.8)**
Phosphorus (mg/1000 kcal)	551 (0.002)	563 (0.002)	12.8 (2.3)***
Iron (mg/1000 kcal)	6.27 (0.002)	6.68 (0.003)	0.41 (6.1)***
Sodium (g/1000 kcal)	0.83 (0.005)	0.89 (0.005)	0.05 (6)***
Potassium (mg/1000 kcal)	1357 (0.002)	1339 (0.002)	17.9 (1.3)***
Copper (mg/1000 kcal)	0.75 (0.012)	0.73 (0.013)	0.01 (2)
Zinc (mg/1000 kcal)	6.18 (0.003)	6.56 (0.003)	0.37 (5.7)***
Selenium (μg/1000 kcal)	50.7 (0.004)	51.6 (0.005)	0.88 (1.7)**
Retinol (μg/1000 kcal)	224 (0.026)	215 (0.029)	8.3 (3.9)
Thiamin (vitamin B1) (mg/1000 kcal)	0.64 (0.002)	0.67 (0.002)	0.03 (3.9)***
Riboflavin (vitamin B2) (mg/1000 kcal)	0.89 (0.003)	0.97 (0.003)	0.09 (9)***
Niacin (vitamin B3) (mg/1000 kcal)	8.31 (0.003)	8.44 (0.004)	0.13 (1.5)
Pyridoxine (vitamin B6) (mg/1000 kcal)	0.84 (0.002)	0.83 (0.003)	0.01 (1.1)***
Cobalamine (vitamin B12) (μg/1000 kcal)	3.23 (0.015)	3.14 (0.017)	0.09 (2.7)**
Folate (dietary equivalent) (μg/1000 kcal)	149 (0.004)	162 (0.004)	12.8 (7.9)***
Vitamin D (calciferol) (μg/1000 kcal)	1.91 (0.008)	1.96 (0.009)	0.05 (2.8)***
Vitamin E (total Alpha-tocopherol) (mg/1000 kcal)	2.28 (0.003)	2.27 (0.003)	0.01 (0.5)
Vitamin C (mg/1000 kcal)	100 (0.027)	109 (0.031)	9 (8.3)**

Means were adjusted for age, gender, region, rural/urban area, and income; **P < 0.01, ***P < 0.001; SE: standard error; Absolute Δ (Difference) Mean Intakes = │Tuber Consumers – Non-Tuber Consumers│ (% Difference)

The diet of those who include tubers contained higher intakes of all macronutrients (protein, carbohydrates, fiber, and fat). The macronutrient differences were mostly attributed to higher energy intake. Among tuber consumers, the energy-adjusted mean protein intake was 4% lower (*P* < 0.001), while the consumption of carbohydrates and fiber was 2 and 0.6% higher (P < 0.001) compared to non-consumers. There was no statistically significant difference in energy-adjusted fat intakes between both consumer types. The micronutrient density of the tuber containing diet was significantly (*P* < 0.01) altered within the Brazilian population, apart from magnesium, copper, retinol, niacin, and alpha-tocopherol remaining unchanged (*P* < 0.1). In particular, the tuber containing diet was found to be less dense in terms of riboflavin (9%), folate (8%), vitamin C (8%), iron (6%), sodium (6%), and zinc (6%) when compared to the diet of non-tuber consumers.

## Discussion

In this study, we investigated the intake of tubers and the prevalence among tuber consumers in rural and urban areas across the five Brazilian macroregions. The percentage of tuber consumption per processing type across the Brazilian macroregions was also examined.

Moreover, this study assessed the differences in tuber consumption between genders and age groups and energy and nutrient intake of tuber consumers versus non-tuber consumers across all Brazil. This study has shown that the prevalence and intake of tubers amongst tuber consumers differed between macroregions, with rural areas having significantly higher mean daily intakes of tubers than urban areas. Compared to women, small but significantly higher tuber intakes were noted for males. There was no significant difference between consumption of tubers among income quintiles. Nutrient intakes between tuber and non-tuber consumers were non meaningfully different, with a few exceptions.

### Tuber consumption across Brazilian macroregions

This study has shown that 55% of the Brazilian population consumed tubers at least on 1 day over the two-day survey. A previous study reported the highest consumption of roots and tubers in rural areas in the South, Southeast, and Central-West [8]. Although the North’s and Northeast’s consumption was well below the national average, rural areas exhibited higher consumption, with some areas meeting or exceeding the national average [8]. This study shows a higher intake of tubers among tuber consumers in rural areas for all five macroregions. The rural area in the North consumed the largest amounts of tubers. The highest consumption of tubers observed in the North’s rural area might be related to mandioca’s highest consumption in that region. In turn, the highest intake of mandioca in the North may be explained by the fact that the North is the largest producer of roots and tubers, of which 87% is mandioca [1].

Mandioca and potato were the most commonly consumed tubers across all Brazil, while the highest daily intakes among tuber consumers were noted for sweet potato and potato. Interestingly, on a macroregion level, the highest prevalence and consumption for sweet potato and potato were observed amongst consumers living in the Northeast and South and the South and Southeast, respectively. The South and Northeast macroregions are the largest sweet potato producers [5], whereby potato production is concentrated in the South and Southeast [9, 10]. The highest intake of mandioca was observed in the North. As discussed earlier, this may be supported by the fact that the North is the largest producer of roots and tubers, of which 87% of the production is mandioca [1].

Mandioca is the most versatile among all tubers consumed in Brazil, and it is incorporated into dishes either cooked whole, in the form of flour, or as refined starch. Typical cooking processes for the whole mandioca include boiling, frying, and roasting. ‘Farinha’ de Mandioca (also referred to as mandioca flour or cassava flour) is an ingredient used to prepare traditional Brazilian dishes such as ‘Farofa’ and ‘Tutu de Feijao.’ Tapioca starch (also called mandioca starch) is a pre-cooked cassava starch with a pearl-like appearance. It is used for making traditional dishes such as ‘Pao de Queijo,’ ‘Biscoito de Polvilho,’ and ‘Brazilian Tapioca’ (crepes) [11–13]. Potato, sweet potato, and yam are other tuber types available on the Brazilian markets for consumption in their whole form [14]. Starch produced from these tubers is not as popular as the starch made from mandioca [15]. This is in parallel with our findings showing that potato, sweet potato, and yam were incorporated into dishes as a whole across all five macroregions. Beet can be consumed as a whole vegetable, juice, chips, and powder [15]. In this study, beet was consumed in dishes either whole or as a juice across all macroregions. Interestingly, the Northeast, which consumed the largest amounts of beet of all macroregions, was also shown to consume the highest proportion of beet juice, accounting for approximately 85% of the total beet consumption in this region.

The carrot consumption didn’t significantly differ across the macroregions and no significant intake differences were observed between urban and rural areas. Nevertheless, a higher prevalence of carrot consumers was noted amongst individuals living in urban areas, with the South, Southeast, and Central-West having the highest scores. Only a small proportion of the carrot produced in Brazil is devoted to processing, i.e., juices, meaning that almost all of the harvest is designated for the fresh market [16]. Not surprisingly, carrots were primarily consumed whole as part of mixed dishes across all of Brazil. Moreover, with the rising awareness of healthy food choices, consumers’ interest in fresh-cut, minimally processed, and ready to eat vegetables is increasing, i.e., the Brazilian mini-carrots, ‘Cenourete’ (shape of a mini carrot root) and ‘Catetinho’ (shape of a small sphere), which can be consumed raw or cooked [16].

### Influence of gender, age, and income on tuber consumption

Male consumers were shown to have higher intakes of tubers than females, although the difference was small. This result is concordant with a previous study demonstrating that men consume more grains, roots, and tubers, and more food in general than women [17]. Concerning the consumers’ age influence on tuber intake, the diet of children 10–12 years of age contained fewer tubers. This is not unexpected, as children’s total energy requirements are lower than for adults, hence the lower consumption of tubers observed in this age group. Nevertheless, the consumption of fruit and vegetables among Brazilian children is insufficient [18]. Moreover, dietary consumption patterns in children are highly influenced by socioeconomic factors [19]. A study on eating habits during childhood has demonstrated that vegetable consumption increases with age [20]. One reason is that children perceive vegetables to be visually unappealing food [21]. The consumption of tubers amongst individuals aged 13 to 18 did not differ from other age groups. This is surprising as adolescents were shown to consume the largest, although still insufficient, amounts of grains, roots, and tubers [17, 22]. Adolescents require more macronutrients than adults as 15 and 40% of the adult height and weight, respectively, are gained during this period [23]. Nevertheless, the expected increase in tuber intakes amongst adolescents was not observed in this study. Differences in mean intakes between income groups across all tuber consumers in Brazil were not found, but there was a trend towards higher daily intakes in lower income quintiles. A previous study supports this result, reporting the lowest intake of grains, roots, and tubers by the Brazilian consumers in the top income category (third tertile) [17].

### Energy and nutrient intake of tuber consumers

The present study showed that approximately 52–53% of the Brazilian diet’s energy intake came from carbohydrates, followed by protein (18%) and fat (12%). Our results agreed with a previous report estimating that up to 57% of Brazil’s total energy intake was attributed to carbohydrates’ consumption [24]. Although Brazil has been a top leader in cassava production, 60% of Brazil’s total carbohydrates intake is coming from rice, beans, bread, coffee, and juices [24]. Our study also found that those who incorporated tubers into their diet consumed 15.5% more energy than non-tuber consumers. The diet of tuber consumers was characterized by having a higher intake of carbohydrates (17%), fats (14.8%), fiber (14.6%), and protein (10.3%) than the diet of non-manusscriptuber consumers (Table 3). A previous report estimated that starchy roots and tubers and manioca flour contributed to 13.2% of the total fiber availability in Brazilian households [25]. After controlling for differences in energy intake and other covariates (Table 3), our findings show that the diet of tuber consuming individuals had 2% more carbohydrate and 4% less protein, and the same amount of fat than the diet of non-consumers. Furthermore, a tuber containing diet was less dense in some vitamins (riboflavin, folate, vitamin C) and minerals (iron, sodium, and zinc) compared to the diet of non-tuber consumers. However, the differences tended to be small (e.g., no relative difference was > 10%).

### Strengths and limitations

The strength of this present study is that it is based on a large nationally representative survey using a rigorous dietary assessment procedure that can allow for flexible secondary analyses. One of the limitations is that a self-report dietary measure is subject to random and systematic reporting errors, which may have unpredictable impacts on tuber consumption estimates and other dietary intake measurements. The sample size was also too small to look at smaller geographies as some patterns may have been concealed at the macroregion level. Another limitation is that the study focused on nutrients and not dietary patterns/food groups, which are likely of more interest as dietary recommendations have shifted towards greater emphasis on foods as opposed to nutrients. Lastly, at the time of the analysis, this was the most up to date data on food consumption in Brazil; data available through FAOSTAT [26] shows a 30% decline in the production of cassava between 2010 and 2019 and a large increase (≈62%) in the production of sweet potatoes during the same period. Lastly, the production of potatoes has almost remained constant between 2010 and 2019. These changes in tuber production may have been driven by consumer demand and may have resulted in changes in the most current amounts and types of tubers consumed by the Brazilian population.

## Conclusions

This study demonstrated that tuber consumption among tuber consumers differed between the Brazilian macroregions, with rural areas having higher intakes than urban. The rural area in the North consumed the largest amounts of tubers, and this may be attributable to the highest production and consumption of mandioca observed in this region. Mandioca is a known staple food in the North and Northeast of Brazil and was consumed as a whole and as flour and starch. Mandioca and potato were the most prevalent amongst tuber consumers across all of Brazil, while the highest intakes among tuber consumers were noted for sweet potato and potato. On a macroregion level, the highest prevalence and consumption for sweet potato and potato were observed amongst consumers living in the Northeast and South (sweet potato) and South and Southeast (potato). These macroregions are the most populated regions concentrating a large proportion of the tuber consumers and are the main producers of these tuber types. Starch produced from potato and sweet potato is not as popular as the starch made from mandioca. This is in parallel with our findings showing that these tuber types were consumed in dishes that incorporated tubers as a whole across all five macroregions. Furthermore, gender and age were shown to influence tuber consumption. A small but significantly higher intake of tubers was noted for male consumers. Tuber consumption resulted in somewhat lower intakes of some micronutrients.

## Supplementary Information

**Additional file 1.**

## Data Availability

Not applicable.
